# Time-Dependent Changes in Microglia Transcriptional Networks Following Traumatic Brain Injury

**DOI:** 10.3389/fncel.2019.00307

**Published:** 2019-08-08

**Authors:** Saef Izzy, Qiong Liu, Zhou Fang, Sevda Lule, Limin Wu, Joon Yong Chung, Aliyah Sarro-Schwartz, Alexander Brown-Whalen, Caroline Perner, Suzanne E. Hickman, David L. Kaplan, Nikolaos A. Patsopoulos, Joseph El Khoury, Michael J. Whalen

**Affiliations:** ^1^Department of Neurology, Brigham and Women’s Hospital, Harvard Medical School, Boston, MA, United States; ^2^Center for Immunology and Inflammatory Diseases, Massachusetts General Hospital, Harvard Medical School, Boston, MA, United States; ^3^Massachusetts General Hospital, Harvard Medical School, Charlestown, MA, United States; ^4^Harvard Medical School, Boston, MA, United States; ^5^Department of Anatomy, Histology and Embryology, School of Basic Medical Sciences, Fudan University, Shanghai, China; ^6^Shanghai Key Laboratory of Medical Imaging Computing and Computer Assisted Intervention, Shanghai, China; ^7^Systems Biology and Computer Science Program, Ann Romney Center for Neurological Diseases, Department of Neurology, Brigham and Women’s Hospital, Boston, MA, United States; ^8^Division of Genetics, Department of Medicine, Brigham and Women’s Hospital, Harvard Medical School, Boston, MA, United States; ^9^Broad Institute of Harvard and Massachusetts Institute of Technology, Cambridge, MA, United States; ^10^Department of Pediatrics, Massachusetts General Hospital, Harvard Medical School, Boston, MA, United States; ^11^Department of Biomedical Engineering, Tufts University, Medford, MA, United States

**Keywords:** traumatic brain injury, microglia, transcriptome, neurodegeneration, mice, neuroimmunology, neuroinflammation

## Abstract

The neuroinflammatory response to traumatic brain injury (TBI) is critical to both neurotoxicity and neuroprotection, and has been proposed as a potentially modifiable driver of secondary injury in animal and human studies. Attempts to broadly target immune activation have been unsuccessful in improving outcomes, in part because the precise cellular and molecular mechanisms driving injury and outcome at acute, subacute, and chronic time points after TBI remain poorly defined. Microglia play a critical role in neuroinflammation and their persistent activation may contribute to long-term functional deficits. Activated microglia are characterized by morphological transformation and transcriptomic changes associated with specific inflammatory states. We analyzed the temporal course of changes in inflammatory genes of microglia isolated from injured brains at 2, 14, and 60 days after controlled cortical impact (CCI) in mice, a well-established model of focal cerebral contusion. We identified a time dependent, injury-associated change in the microglial gene expression profile toward a reduced ability to sense tissue damage, perform housekeeping, and maintain homeostasis in the early stages following CCI, with recovery and transition to a specialized inflammatory state over time. This later state starts at 14 days post-injury and is characterized by a biphasic pattern of IFNγ, IL-4, and IL-10 gene expression changes, with concurrent proinflammatory and anti-inflammatory gene changes. Our transcriptomic data sets are an important step to understand microglial role in TBI pathogenesis at the molecular level and identify common pathways that affect outcome. More studies to evaluate gene expression at the single cell level and focusing on subacute and chronic timepoint are warranted.

## Introduction

Traumatic brain injury (TBI) is a leading cause of mortality, morbidity, and disability in children and adults, with an economic burden of over $60 billion per year in the United States (Langlois et al., 2006; McKinlay et al., 2008). No specific therapy is proven to reduce long-term cognitive sequelae of TBI and only limited options exist for rehabilitation (Gordon et al., 2006; McCrory et al., 2009; Helmick and Members of Consensus Conference, 2010), in part because mechanisms driving injury and outcome remain poorly defined. One mechanism thought to be important in long-term outcome after TBI is neuroinflammation (Ramlackhansingh et al., 2011; Loane et al., 2014; Jassam et al., 2017; Simon et al., 2017a). TBI activates resident microglia, induces cytokines production in the brain, and causes influx of peripheral immune cells, followed by a chronic activation of resident microglia and astrocytes (Das et al., 2012; Sofroniew, 2015; Simon et al., 2017a). Neuroinflammation can participate in repair mechanisms and has also been proposed as a potentially modifiable driver of secondary injury in animal and human studies (Jassam et al., 2017; Simon et al., 2017b). However, the precise cellular and molecular mechanisms of neuroinflammation leading to neurological deficits at acute, subacute, and chronic time points after TBI remain to be defined (Jassam et al., 2017; Simon et al., 2017a).

Microglia are macrophage-like cells that reside in the central nervous system (CNS) and play a critical role in neuroinflammation (Ransohoff and El Khoury, 2015; Hickman et al., 2018). They are among the first responders to brain injury and their persistent activation in TBI animal models and in humans with TBI may contribute to long-term functional deficits (Gentleman et al., 2004; Coughlin et al., 2015; Witcher et al., 2015; Muccigrosso et al., 2016; Jassam et al., 2017). Activated microglia may be characterized based on morphological transformation from ramified (resting state) to amoeboid (activated state) (Donat et al., 2017; Hickman et al., 2018). Alternatively, transcriptomics can be used to describe specific inflammatory states (Hickman et al., 2018). Transcriptomic analyses of microglia in aged mice and human macrophages showed that these cell types undergo disease and stimulus-specific gene expression changes (Hickman et al., 2013; Xue et al., 2014). Our understanding of microglia-specific gene functions, pathways, and networks in Alzheimer disease (AD), amyotrophic lateral sclerosis, and other neurodegenerative diseases has been rapidly evolving and now offers a road map to study gene expression in other complex diseases like TBI (Chiu et al., 2013; Hickman et al., 2013, 2018).

Despite the evidence for a significant role for microglia in the pathogenesis of TBI, few studies to date have examined microglia-specific gene expression as a function of time in a preclinical TBI model. Almost all prior studies in preclinical TBI models used whole brain tissue to infer inflammatory responses to injury of microglia and other brain cell types (Kobori et al., 2002; Matzilevich et al., 2002; Almeida-Suhett et al., 2014; Lipponen et al., 2016); however, this approach does not necessarily reflect microglia-specific gene expression changes (Hickman et al., 2013). Moreover, most of these prior studies were limited to acute post-injury time points (Redell et al., 2013; Samal et al., 2015; White et al., 2016; Wong et al., 2016). A recent single-cell RNA sequencing study of cells isolated by fluorescence activated cell sorting (FACS) from mouse TBI brain samples at 24 h after fluid percussion injury provided unique information, based on only 249 cells from sham and 293 cells from injured animals, about how TBI impacts diverse gene expression changes in hippocampal cell types (Arneson et al., 2018). However, to our knowledge, no study has characterized temporal expression patterns, including the chronic period, of microglia-specific inflammatory gene expression in a preclinical TBI model.

Here, we analyzed the temporal course of changes in inflammatory gene transcription of microglia isolated from injured brain by FACS up to 60 days after controlled cortical impact (CCI) in mice, a well-established model of focal cerebral contusion (Jassam et al., 2017), using the Nanostring gene expression analysis platform. We identified a time-dependent, injury-associated change in the microglial phenotype toward a reduced ability to sense tissue damage, perform housekeeping, and maintain homeostasis in the early stages following CCI, with recovery and transition to a pro-inflammatory state over time.

## Materials And Methods

### Experimental Animals

Studies were performed using 3-month-old male C57BL6J mice (Stock #000664, Jackson Laboratories, Bar Harbor, ME, United States). All procedures were performed in accordance with the NIH Guide for Care and Use of Laboratory Animals and followed protocols approved by the MGH Institutional Animal Care and Use Committee. Mice had access to food and water *ad libitum* and were housed on a 12-h day–night cycle in laminar flow racks in a temperature-controlled room (25°C). Investigators were blinded to study groups in all experiments. Mice were randomized to sham and injured at 2 days post-injury (dpi). Shams and CCI mice were housed in the same cage. Subsequent groups were injured at 14 and 60 dpi without any specific randomization scheme since the mice were genetically identical and compared to 2 day shams.

### Controlled Cortical Impact (CCI)

A CCI model was used as previously described (Bermpohl et al., 2007). Mice were anesthetized with 4.5% isoflurane (Anaquest) in 70% nitrous oxide and 30% oxygen using a Fluotec 3 vaporizer (Colonial Medical). The mice were placed in a stereotaxic frame and a 5-mm craniotomy was made over the parieto-temporal cortex using a drill and a trephine. The bone flap was removed and discarded, and a pneumatic cylinder with a 3-mm flat tip impounder with velocity 6 m/s, depth 0.6 mm, and duration 100 ms was used to induce CCI. The scalp was sutured closed and the mice were returned to their cages to recover.

### Preparation of Brain Tissue for CD11b Immunohistochemistry

The mice were anesthetized with avertin and transcardially perfused with PBS. The brains were extracted and postfixed in 4% paraformaldehyde overnight, cryoprotected in 15% followed by 30% sucrose overnight, frozen at −80°C, prior to making coronal sections (12 μm) on poly-L-lysine-coated slide (Thermo Fisher Scientific) using the cryostat. The brains were cut at 0.5 mm intervals from the anterior to the posterior of the brain, starting at 1.56 mm from the bregma position.

### CD11b Immunohistochemistry

Brain sections were washed twice with Dulbecco’s phosphate-buffered saline (DPBS) (without calcium and magnesium) before undergoing fixation with 95% ethanol (pre-cooled to 4°C) for 10 min. Antigen retrieval was performed with a 15-min incubation of pre-warmed (37°C) trypsin (0.25%) before blocking endogenous peroxidase activity with a 5-min incubation of 0.3% hydrogen peroxide and 0.3% normal rabbit serum in DPBS. Sections were blocked in DPBS with 1.5% normal rabbit serum for 20 min before primary antibody incubation with 1:100 CD11B (Serotec MCA711G; Serotec, Oxford, United Kingdom) in DPBS with 2.5% normal rabbit serum. The primary antibody was visualized with the avidin–biotin horseradish peroxidase technique in accordance with manufacturer’s instructions [Vector Laboratories, Vectastain Elite Kit PK6104, NovaRED Peroxidase (HRP) Substrate Kit SK-4800, Burlingame, CA, United States]. Sections were then counterstained with hematoxylin in accordance with the manufacturer’s instructions (Vector Laboratories, H-3401, Burlingame, CA, United States), and mounted with Vectamount Permanent Mounting Medium (Vector Laboratories, H-5000, Burlingame, CA, United States). Brightfield images were subsequently obtained using a Zeiss Axio Scan.Z1 slide scanner with the ZenBlue software (Carl Zeiss, Jena, Germany).

### CD11b+ Surface Area Quantification

**FIGURE 1:**
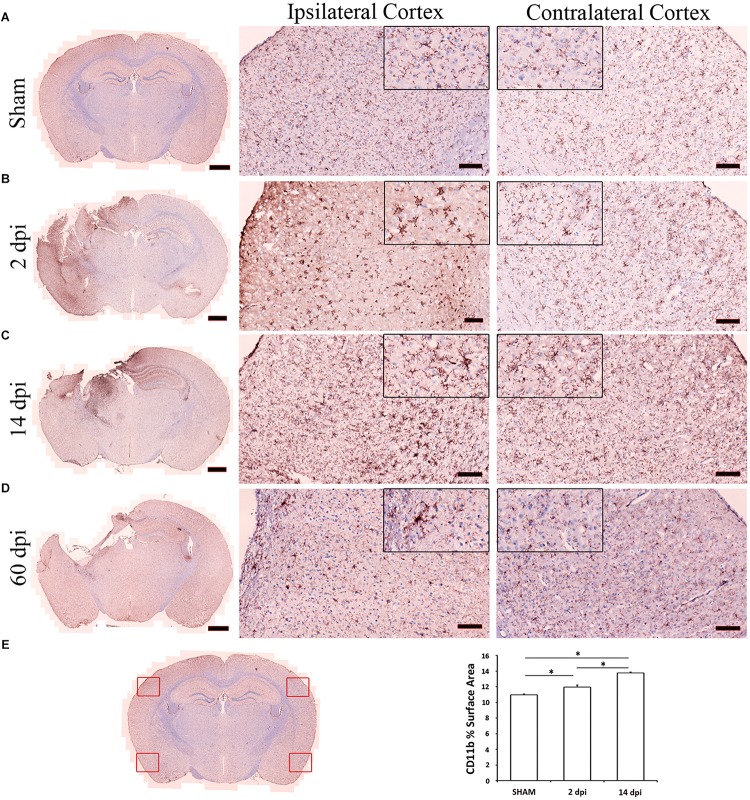
Microglia activation after controlled cortical impact (CCI). Microglia were labeled with anti-CD11b antibody. This figure shows representative photomicrographs of cortical microglia taken from contralateral and ipsilateral hemispheres from sham **(A)**, 2 **(B)**, 14 **(C)**, 60 **(D)** days post-injury (dpi). The left column illustrates the distribution of microglia activation at low magnification. Compared to the uninjured sham **(A)**, increased CD11b staining is observed across the cortex at 2 **(B)** and 14 dpi **(C)**. Highly ramified microglia with spherical cell bodies in sham animals become less ramified with swollen or stretched cell bodies by 2 and 14 dpi (see magnified 20 μm corner views). Images are representative of three animals per time point. Scale bars are 1 mm, 100 μm, and 20 μm for photomicrographs on increasing magnification. **(E)** Scanned photomicrograph was used to produce four images as representative of each hemisphere and two from the contralateral hemisphere. CD11b surface area was analyzed using ImageJ software and quantification data are shown as mean ± SEM differences in CD11b surface area between region and time post-injury. Compared to the uninjured sham, there is significant increase in CD11b surface area across the cortex at 2 (*p* = 0.0006) and 14 dpi (*p*< 2*e*–16).

The presence of microglia was determined by quantifying CD11B+ cell surface area in the cortex of coronal brain sections stained slides. Analysis of percent microglia-positive surface area was performed on 10 photomicrographs per animal (*n* = 3 mice for each time point). Each scanned photomicrograph was used to produce four images: two from the ipsilateral hemisphere and two from the contralateral hemisphere. Within the ipsilateral hemisphere, one image was taken in the cortex, peripheral to the site of injury, while one image was taken far from the site of injury. The two contralateral hemisphere images mirrored the locations of the ipsilateral images. Together, these images were taken as representative of each hemisphere (Figure 1E). Each of the four images was analyzed using ImageJ software (National Institute of Health^1^). Images were split by color channel, and the channel of interest was thresholded using the Yen setting. ImageJ was used to analyze the percent of surface area occupied by CD11B+ cells in each image. CD11b quantification data are shown as mean ± SEM and analyzed using R studio with statistical significance assigned when *p* < 0.05. Differences in CD11b surface area between 2 and 14 dpi vs. sham were analyzed using a one-way analysis of variance (ANOVA) followed by a Tukey’s honest significant difference test.

### Isolation of Brain Microglia by Fluorescence Activated Cell Sorting (FACS)

Microglia were isolated by FACS as previously described (Hickman et al., 2013). The mice were anesthetized with avertin and transcardially perfused with PBS. The brains were extracted and placed in PBS on ice. The brains were dissociated using collagenase and dispase and gentleMACS dissociator (Miltenyi Biotech). The cells were separated using physiologic Percoll and were blocked with donkey serum and fetal bovine serum and incubated with APC anti-mouse CD11b and PerCP anti-mouse CD45 antibodies for 30 min. Microglia were sorted based on CD45 low to intermediate/CD11b high expression using BD FACSAria II (Becton Dickinson, Inc.) (Supplementary Figure S1).

### Nanostring Gene Expression Analysis

Microglia isolated by FACS were stored in RNAlater Stabilization Solution (AM7020, Invitrogen) at −80°C until further processing. Total RNA from the sorted cells was extracted using miRNeasy Micro Kit from Qiagen (Redwood City, CA, United States) for purification of total RNA from small amounts of cells according to the manufacturer’s instructions. Microglia RNA integrity number (RIN) was between 8 and 9. mRNA expression analysis with the NanoString nCounter^®^ Mouse Inflammation v2 Panel was performed according to the manufacturer’s protocol (NanoString Technologies Inc., Seattle, WA, United States).

### Quantitative Real-Time Polymerase Chain Reaction

Relative expressions of selected microglia genes for sham vs. 2 dpi were verified by real-time qPCR. First-strand complementary DNA (cDNA) was synthesized using RT2 First Strand Kit (Qiagen, Redwood City, CA, United States) according to manufacturer’s instructions. RT^2^ qPCR Primer Assay for Mouse IL-1β, IL-6, and TNF (Qiagen, Redwood City, CA, United States) were used according to manufacturer’s protocol to validate the gene expression changes shown by Nanostring. Data normalization was performed by quantification of the endogenous 18S ribosomal RNA.

### Bioinformatics

#### Data Normalization

The raw Nanostring nCounter genes were normalized using NanoStringDiff (Wang et al., 2016), adjusting for positive controls, housekeeping genes, and background level. The NanoStringNorm (Waggott et al., 2012) packages was used to generate normalized gene expression, for plotting and clustering purposes.

#### Differential Gene Expression Analysis

We use a generalized linear model (GLM) with negative binomial family as implemented in the NanoStringDiff (Wang et al., 2016) to examine differential gene expressions for the following comparisons: acute (2 dpi vs. sham), subacute (14 dpi vs. sham), and chronic (60 dpi vs. sham).

#### Pattern-Oriented Time-Series Clustering

To better model and discover genes that follow time-series patterns that are of potential biological interest, we developed a framework named pattern-oriented time-series clustering. Firstly, the gene expressions normalized by NanoStringNorm were filtered by 25% quantile in terms of mean and standard deviation. The rest of the genes were standardized between 0 and 1. Secondly, we calculated the differences in the standardized gene expression for the following pairwise comparisons: 2 dpi vs. sham, 14 dpi vs. 2 dpi, and 60 dpi vs. 14 dpi, and named the differences standardized linear changes (SLCs). We used one group of sham (*n* = 4) as a baseline for comparison with the three other time points including 2 (*n* = 4), 14 (*n* = 4), and 60 dpi (*n* = 3). We used SLC to depict the temporal patterns that could not be captured by either *p*-value or log2 (fold-changes) alone. We defined four sub-patterns, namely up, down, stable, and noisy, to compose the time-series pattern for a certain gene. Up and down are the trends with relatively large up or downward SLCs, signaling the genes and intervals with the most changes, while stable are the trends with the lowest absolute value of SLCs, representing the genes that are most stable during such intervals. The trends are categorized as noisy otherwise.

To decide the sub-pattern of each time interval, for each gene, the time interval with the largest absolute SLC, *|*SLC_*max*_*|*, was named up/down by the sign of SLC. Then we calculated absolute relative SLC, *|*SLC_*i*_/SLC_*max*_*|*, where *i* is the index to one of the other two intervals. To determine those sub-patterns, we defined two parameters, cutoff_*up*_ and cutoff_*low*_. If the absolute relative SLC was greater than cutoff_*high*_, the corresponding sub-pattern was categorized as up/down according to the sign of SLC; if less than cutoff_*low*_, it was categorized as stable; otherwise, it was categorized as noisy. From this analysis there were 4^4^ = 64 possible patterns, i.e., clusters of genes. We performed sensitivity analyses for the two cutoff parameters, tuning cutoff_*high*_ from 0.3 to 0.75, by 0.05, and cutoff_*low*_ from 0.05 to 0.275, by 0.025, and accept parameter based on *p*-measurement, defined as *p*_measurement = *w*/*c* × *K*_1_ × *K*_2_, where *c* is a constant, *w* is the weight defined as *w* = 1/(cutoff_*high*_ × cutoff_*low*_) to penalize on selecting loose cutoffs (Supplementary Figure S2). *K*_1_ and *K*_2_ are the number of clusters with no noisy sub-patterns, and number of clusters with no opposite-directed sub-patterns, respectively. The latter was used to select patterns that are only biologically meaningful to our subjects.

#### Gene Set Enrichment and Pathway Analyses

We performed gene set enrichment analysis (GSEA) (Subramanian et al., 2005) on the normalized gene expression dataset with REACTOME and KEGG datasets from c2.all.v6.2.cymbols.gmt curated gene sets using 1,000 permutations. Pathways with false discovery rate (FDR) < 0.05 were selected as significant. We plotted heatmap for the gene sets from cytokine regulated genes as defined by Xue et al. (2014). The genes in heatmap were clustered by *k*-means clustering methods with *k* selected using the gap-statistics.

#### Protein–Protein Interaction Networks

To identify the top regulators in a certain cluster of genes, we performed network analysis using STRING database (Szklarczyk et al., 2017) with all default settings. The top regulators were selected by ranking the sum of confident scores greater than 0.4 (by default) of each gene.

## Results

**FIGURE 2:**
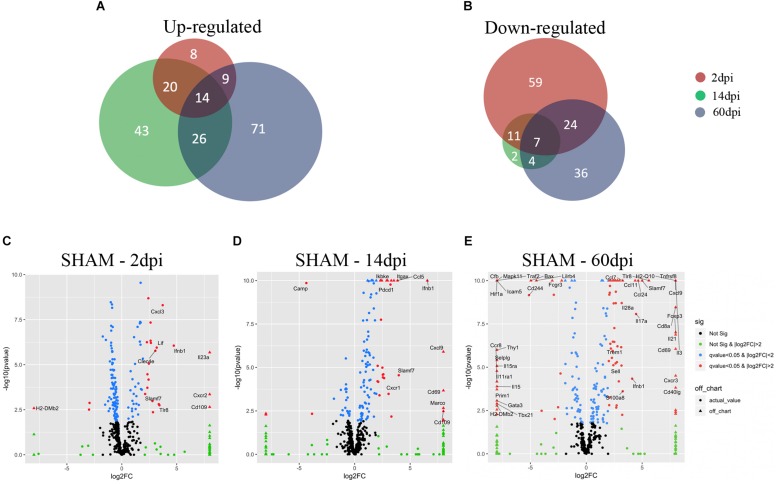
Microglial gene expression after CCI. **(A,B)** Venn diagrams showing the overlap between 2, 14, and 60 dpi, vs. sham of **(A)** up-regulated genes (adjusted *p*-value < 0.05) and **(b)** down-regulated genes (adjusted *p*-value < 0.05). **(C–E)** Volcano plot showing the microglia gene expression of **(C)** 2 dpi vs. sham, **(D)** 14 dpi vs. sham, and **(E)** 60 dpi vs. sham. On the *x*-axis are the log2-fold changes and the *y*-axis is the –log10(*p*-value). Genes in black: False discovery rate (FDR) ≥ 0.05, |log2(FC)| ≤ 2; genes in green: FDR ≥ 0.05, |log2(FC)| > 2; genes in blue: FDR < 0.05, |log2(FC)| ≤ 2; and genes in red: FDR < 0.05, |log2(FC)| > 2. Genes in red with FDR ≤ 0.05, |log2(FC)| > 3 are named.

We subjected 2-month-old male C57BL6J mice (Jackson Laboratories, *N* = 3–4/group) to either CCI or to sham injury using established protocols (Bermpohl et al., 2007). CCI produced a cavitary lesion and overt hippocampal damage associated with morphological changes in microglia indicative of activation (Fox et al., 1998). Figure 1 shows representative photomicrographs of cortical microglia taken from contralateral and ipsilateral hemispheres at 2–60 dpi. CCI caused a significant increase in microglia numbers at 2 and 14 dpi vs. sham, and clear morphological changes consistent with microglia activation at 2, 14, and 60 dpi in ipsilateral cortex including shorter, thicker, and less ramified processes and swollen or stretched cell bodies. Similar changes in microglia morphology were also observed in some contralateral regions (Figures 1A–D). Microglia were isolated from brain tissue by enzymatic digestion as previously described (Hickman et al., 2013). Cells were stained with fluorescent antibodies to CD11b and CD45, two well-established microglia and macrophage markers (Sedgwick et al., 1991) and subsequently isolated using FACS at 2, 14, and 60 dpi as previously described (Sedgwick et al., 1991). RNA was then isolated from microglia and subjected to Nanostring analysis, a quantitative gene expression analysis platform with qPCR sensitivity using a probe set of 550 genes involved in inflammation and immunity (O’Neil et al., 2018; Zhang et al., 2018). We found more genes upregulated significantly at 60 dpi than 2 or 14 dpi (Figure 2A).

### Microglia Gene Expression at 2 Days Post-injury

**FIGURE 3:**
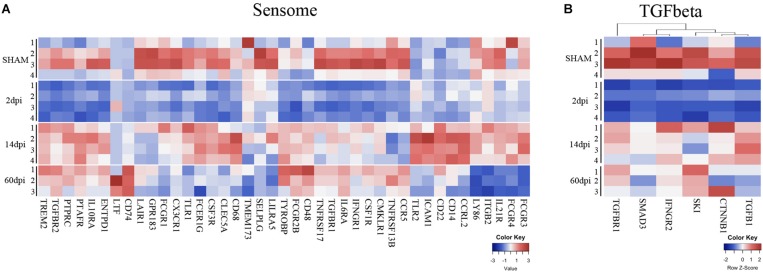
Time-dependent changes of microglial sensome genes and TGF-beta pathway after TBI. Heatmap of normalized gene expressions profiles for **(A)** microglia sensome and **(B)** TGF beta signaling pathway.

**FIGURE 4:**
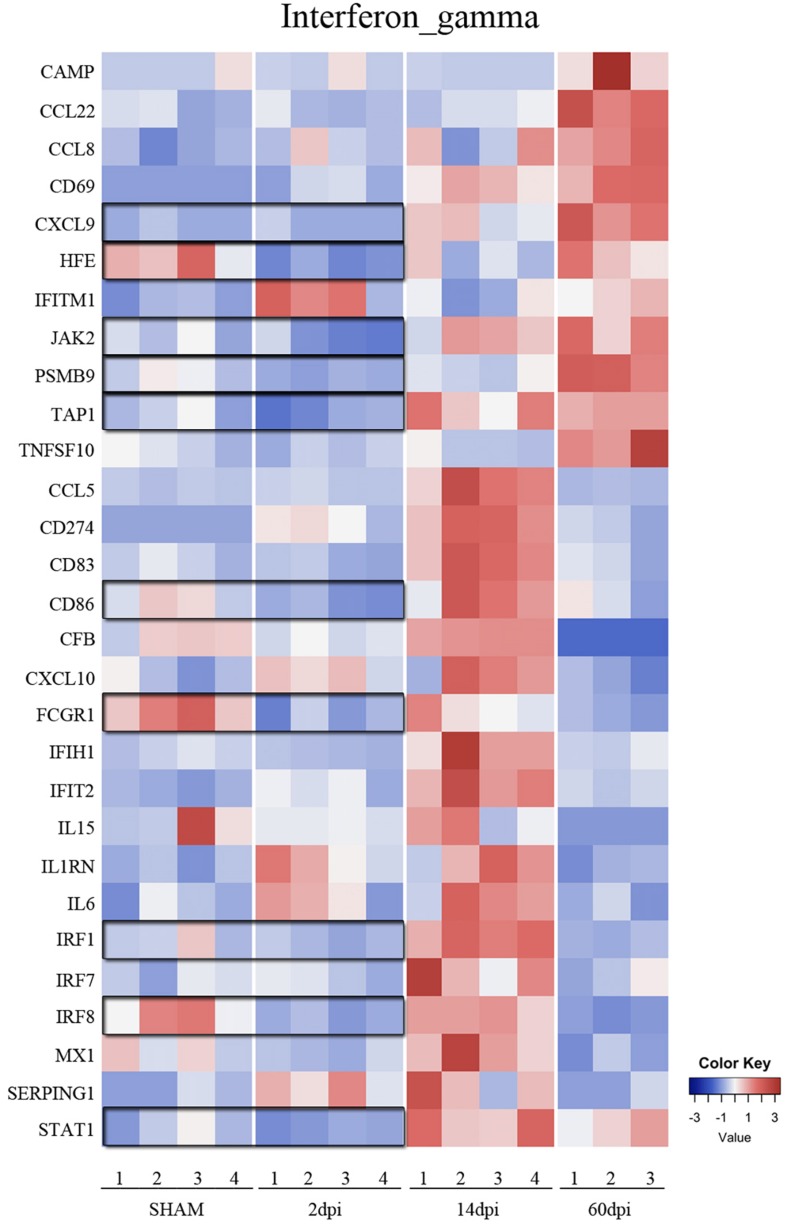
Time-dependent changes of interferon gamma signaling pathway after TBI. Heatmap of genes in the interferon gamma signaling pathway at 2, 14, and 60 dpi vs. sham.

Using the Nanostring^TM^ gene expression platform, we identified 152 genes with significant changes in expression at 2 dpi compared to sham, including 51 upregulated and 101 downregulated genes (Figures 2A,B and Supplementary Table S1). Genes associated with chemotaxis (CXCL1, CXCL3, CCL19, CXCR1, CXCR2, CXCR4, CCR2) as well as cytokine signaling (IL1b, IL1R2, IL23a, IL6, TNF, and IFNB1) were significantly upregulated, suggesting a role for microglia in recruitment of microglia from neighboring areas of the brain as well as recruitment of peripheral leukocytes to injured brain (Liu et al., 2018). Genes involved in the anti-inflammatory transforming growth factor-beta (TGF-β) cytokine signaling pathway (TGFB1, TGFBR1, TGFBR2, SMAD3, SKI) were significantly downregulated at 2 dpi (Figure 3B). Likewise, subset of genes from the Interferon gamma (IFNγ) pathways (HFE, TAP1, JAK2, STAT1, CD86, FCGR1, PSMB9, IRF8, IRF1, CXCL9) were also downregulated at 2 dpi (Figure 4).

IL23a was one of the most significantly upregulated genes in 2 dpi (Figure 2C and Supplementary Table S1). IL23a is a cytokine required for the expansion and survival of TH17 cells that also drives a pathogenic T cell population that induces inflammation (Aggarwal et al., 2003). The exact impact of IL23a upregulation is not clear. We also observed significant upregulation in CXCR2 gene expression, a chemokine that regulates neutrophil migration in a closed head injury model that may also play a role in secondary brain damage (Semple et al., 2010) (Figure 2C and Supplementary Table S1). The third most induced gene was CD109, which is known to modulate TGF-β receptor endocytosis and degradation and thereby play a critical role in inhibiting TGF-β signaling (Bizet et al., 2011; Figure 2C and Supplementary Table S1). We also found increased pro-inflammatory IL1 beta as well as CXC chemokine ligand 1 (CXCL1), a chemoattractant for neutrophils (Szmydynger-Chodobska et al., 2016; Arneson et al., 2018; Supplementary Table S1). Our findings at 2 dpi are consistent with a recently published single-cell RNA sequencing study which evaluated the impact of acute (1 dpi) fluid percussion injury on cell type-specific genes and found a significant increase in IL-1 beta and CXCL1, and Cebpb (Arneson et al., 2018). Together, the downregulation of IFNγ and TGF-β cytokine pathways and concomitant upregulation of pro-inflammatory genes suggest a heterogenous inflammatory, stimulus-specific microglial activation state rather than a predominant proinflammatory “M1” or anti-inflammatory “M2” state as previously described for classical macrophage activation (Mills et al., 2000; Morganti et al., 2016b).

### Microglia Gene Expression at 14 Days Post-injury

We identified 127 genes with statistically significantly altered expression in the subacute phase, including 103 upregulated and 24 downregulated vs. sham (Figures 2A,B and Supplementary Table S2). Similar to the 2 dpi findings, many upregulated genes were involved in chemokine signaling (CCL3, CCL4, CCL5, CCL8, CCL19, CXCL1, CXCL9, CXCL10, CXCL13, CXCR1, CXCR4) and proinflammatory cytokine signaling. Interferon gamma pathway genes including IRF1, IRF7, JAK2, MX1, STAT1, TAP1, CCL5, CCL8, CD274, CD69, CD86, CD83, CXCL10, CXCL9, lFIH1, LFIT2, IL1RN were significantly upregulated at 14 dpi compared to sham. Expression of TGF-β signaling genes, which were significantly downregulated vs. sham at 2 dpi, appeared to increase at 14 dpi compared to 2 dpi. In addition, CD109 continued to be one of the highest upregulated genes at this subacute timepoint (Bizet et al., 2011) even though TGFβ pathway genes were down (Figure 2D and Supplementary Table S2).

At 14 dpi, macrophage receptor with collagenous structure (Marco), a scavenger receptor on macrophages that regulates phagocyte innate immune responses, was one of the most significantly upregulated genes (Jing et al., 2013; Figure 2D and Supplementary Table S2). The complement system is another key mediator of the systemic innate inflammatory response which participates in many functions, including opsonization, phagocytosis, immune cell chemotaxis, and cell lysis, among others (Hammad et al., 2018). We found significant increases in gene expression of complement components C1s, C3, and C4a, which is consistent with an important role for complement at the subacute time point (Yager et al., 2008; Alawieh et al., 2018; Hammad et al., 2018). These data suggest increased capacity for phagocytosis at 14 dpi, possibly to promote debris clearance caused by injury. Interestingly, we also found significant upregulation of CD69, which is an immunomodulatory molecule induced during lymphocyte activation, deficiency of which has been recent found to associate with poor outcome after ischemic stroke (Brait et al., 2019; Figure 2D and Supplementary Table S2).

DNA Primase Subunit 1 (Prim1) and Cxcl3 were among the most downregulated genes at 14 dpi (Supplementary Table S2). Cxcl3 plays a key role in promoting neutrophil migration across epithelial barriers (Szmydynger-Chodobska et al., 2009; Liu et al., 2018). The finding that Cxcl3 is downregulated by 14 dpi is consistent with reduction of neutrophil infiltration at 7 days after CCI in rodents (Clark et al., 1994). Downregulation of a cyclic AMP (cAMP) pathway in the acute stages post-injury also persisted to 14 dpi (Atkins et al., 2007; Figure 2D). Restoration of cAMP levels in a fluid-percussion injury and other models of CNS injury was associated with improved functional outcome (Block et al., 1997; Nikulina et al., 2004; Atkins et al., 2007). Together, our findings suggest a dynamic gene expression response to CCI in microglia that is apparent at 14 dpi and includes reinstating TGF beta and IFNγ pathway gene expression as well as induction of phagocytosis pathways including complement.

### Microglia Gene Expression in the Chronic Phase Post-injury (60 Dpi)

In the chronic phase post-injury, we observed 191 genes significantly changed, including 120 genes that were upregulated and 71 downregulated compared to sham (Figures 2A,B and Supplementary Table S3). Upregulated genes in the chronic phase were involved in adaptive and innate immune pathways as well as cytokine and chemokine signaling, suggesting an evolution toward a pro-inflammatory state in the chronic period (Supplementary Table S3). For instance, compared to sham, many genes in the IFNγ cytokine signaling pathway were significantly upregulated compared to sham at 60 dpi (Figure 4).

Il-3, a cytokine which stimulates proliferation of myeloid lineage cells was one of the most induced genes at 60 dpi (Figure 2E). Interestingly, injection of Il-3 and granulocyte macrophage colony stimulating factor together from 2 to 7 days after stab wound injury in rats attenuates brain tissue loss and improves motor function at 2 months, suggesting a reparative function for increased IL-3 (Nishihara et al., 2011). Tumor necrosis factor receptor superfamily member 8 (Tnfrsf8) was also one of the most significantly upregulated genes at 60 dpi, and is known to regulate the proliferative potential of autoreactive CD8 effector T cells and protect the body against autoimmunity (Oflazoglu et al., 2009; Figure 2E). We also found significant upregulation of IL-21 (Figure 2E). IL-21 is known to regulate immune responses by promoting antibody production, T cell-mediated immunity, and NK cell and CD8^+^ T cell cytotoxicity; inhibiting IL-21 in experimental stroke correlated with improved behavioral outcome (Clarkson et al., 2014).

Several TBI studies observed significant upregulation of hypoxia-inducible transcription factor-1α (HIF-1α) acutely post-injury (Ding et al., 2009; Huang et al., 2010), we found significant downregulation of HIF-1*α* at 60 dpi (Figure 2E and Supplementary Table S3). HIF-1α upregulates genes involved with blood–brain barrier (BBB) disruption (Yan et al., 2011), edema formation (Higashida et al., 2011), and apoptosis (Bruick, 2000). A recent study in a closed head injury model showed that the inhibition of HIF-1α was associated with worse motor deficit, hypothermia, and increased lesion size, suggesting a role for HIF-1α in recovery after TBI (Umschweif et al., 2013). The correlation between HIF-1α downregulation at 60 dpi and long-term outcome cognitive deficits after CCI may suggest a functional role for HIF-1α in the chronic period.

### Microglial Gene Expression Over the Course of CCI

In order to identify genes that follow a specific pattern over the three study time points, we performed pattern-oriented time-series modeling and clustering. According to pairwise comparisons, there were 191 genes with significant time-dependent changes when we compared sham vs. 60 dpi (FDR < 0.05). Focusing on genes that were changed at 60 dpi, we built a clustering model to study their expression patterns over time (e.g., compared to 2 and 14 dpi). We found 13 meaningful clusters; 10 clusters had less than 5 genes, and 3 clusters with more than 5 genes are described below (Supplementary Table S4 and Supplementary Figure S3).

The first cluster included 52 genes which were stable at 2 and 14 dpi and significantly upregulated at 60 dpi. Genes in this cluster were involved in secondary injury mechanisms including cytokine receptor and cytokines interactions, chemokine receptors, and immune, adaptive immune system, and cell-mediated toxicity. The second cluster included eight genes which were stable during acute but became significantly upregulated over the subacute and chronic time points post-injury. Two genes were involved in INF gamma signaling including (IRF3 and VCAM) and two other genes in hemostasis including (CLU, CD48). TNFSF12 and CD5 were also upregulated in this manner. The third cluster included five genes which were stable during acute and subacute time points and became significantly downregulated at chronic stages post-injury. Three genes involved in apoptosis were FADD, BAX, PSMB7, and two others involved in complement activation including ITGB2 (integrin, beta 2) (a complement component 3 receptor 3 and 4 subunit) as well as C1QBP (complement component 1, q subcomponent binding protein) (Supplementary Figure S3).

### Changes in Microglia Sensome Genes After CCI

One of the major functions of microglia is sensing changes in the brain environment including injury signals (Hickman et al., 2018). Our group has previously identified 100 genes involved in this process that constitute what we termed the microglia “sensome” (Hickman et al., 2013). To determine the effects of CCI on the microglial sensome, we analyzed the pattern of changes of the sensome genes in response to CCI. Sensome genes that were significantly changed by CCI in a unique and time-dependent pattern (Figure 3A). Of the 46 sensome genes examined, we found the majority of sensome genes downregulated at 2 dpi with statistically significant downregulation of 21 of these genes and only 2 genes (Clec5a and Tlr2) that were significantly upregulated. Sensome gene expression began to normalize at 14 dpi with only some sensome genes that were upregulated (Tlr2, Cd86, Tyrobp, Ptafr, Icam1, Icam4, Ccrl2, Cd14, CD22, CD48, CD74) and five genes were significantly downregulated (Selplg, Gpr183, Ccr5, Lair1, Tnfrsf13b). The trend continued at 60 dpi, where only five sensome genes remained upregulated (Tyrobp, Cd74, Ltf, CD22, CD48) (Figure 3A). Sensome genes, like TREM2, (triggering receptor expressed on myeloid cells 2), which play a key role in transforming microglia from a homeostatic to a neural disease-associated state was also found to be downregulated at 2 dpi followed by upregulation at later time points (Hickman and El Khoury, 2014; Figure 3A).

Together, the data suggest a biphasic pattern of changes in the sensome genes represented by initial downregulation at 2 dpi followed by return to baseline of the majority of these genes at 14 and 60 days (Figure 3A). These changes suggest that there may be dysregulation in ability of microglia to sense tissue damage, perform housekeeping, and maintain hemostasis in the early stages after CCI with possible subsequent recovery over time.

### Cytokine Gene Expression Signatures After CCI

To determine the time-dependent effect of TBI on the microglia cytokine pathways, we mined our gene set for cytokine regulated genes as defined by Xue et al. (2014). As we observed with sensome genes, we also found a time-dependent change in genes involved in cytokine pathways following TBI. Interestingly, in parallel to changes in the sensome changes, we found downregulation of genes involved in the TGFβ pathway at 2 dpi (Figure 3B) TGF-β is required for maintaining the microglial homeostatic state (Butovsky et al., 2014) and is likely needed for maintaining expression of sensome genes. Our data suggest that downregulation of sensome genes at 2 dpi is possibly linked to downregulation of the TGFβ pathway at this early time point post-injury.

**FIGURE 5:**
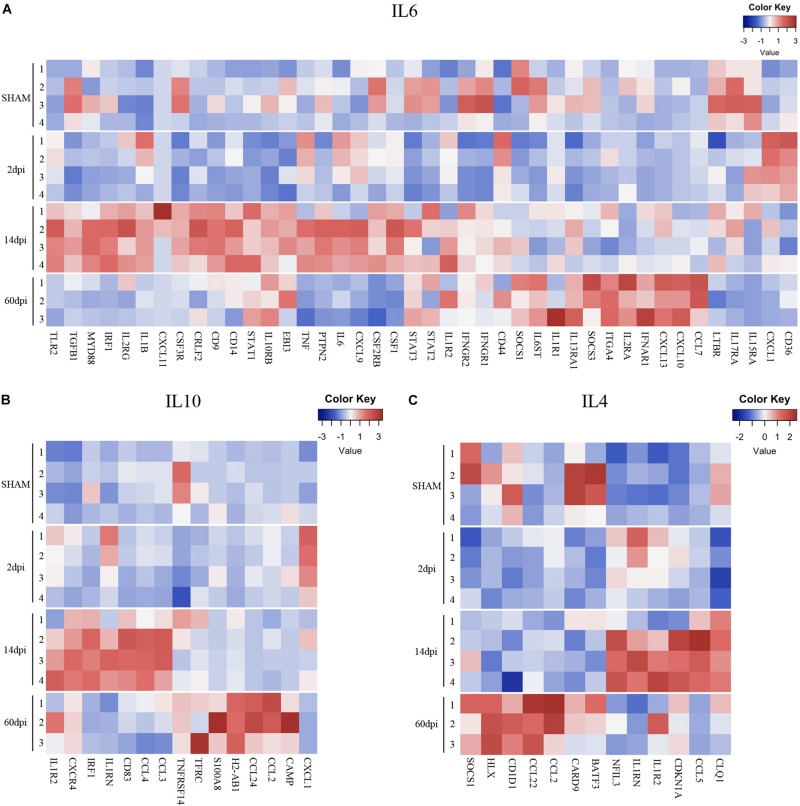
Pro- and anti-inflammatory cytokine gene expression profiles after TBI. Heatmap of normalized gene expressions profiles for **(A)** IL6, **(B)** IL10, and **(C)** IL4 signaling pathways.

In contrast to sensome and TGFβ pathway genes, genes from various cytokine pathways were mostly upregulated at 14 dpi. These include genes from pro- and anti-inflammatory pathways such as IL-6, Interferon-gamma (proinflammatory), and IL-4 and IL-10 (anti-inflammatory) indicating a mixed pattern of gene regulation (Figures 4, 5) that participate in injury and repair mechanisms. Our data are consistent with other TBI studies which suggest that the role of microglia in the CCI model is more complex than the oversimplified prior classification of M1 vs. M2 (Kumar et al., 2016; Morganti et al., 2016b). This mixed state appears to persist at 14 and 60 dpi.

Interestingly, a pattern of regulation of cytokine pathways genes including IL-10, IL-4, interferon gamma, and IL-1 emerged. Genes upregulated at 14 dpi returned to baseline at 60 dpi, whereas genes that remained stable at 14 dpi were upregulated at 60 dpi (Figures 4, 5). Such pattern suggests a defined sequence of gene expression changes post-TBI.

### Potential Regulators of Gene Networks in TBI

To identify known and predicted functional protein–protein interactions relevant to the microglia transcriptome after CCI, we used the STRING v11.0 database (von Mering et al., 2003). STRING quantitatively integrates protein interaction data from multiple sources for a large number of organisms.

**FIGURE 6:**
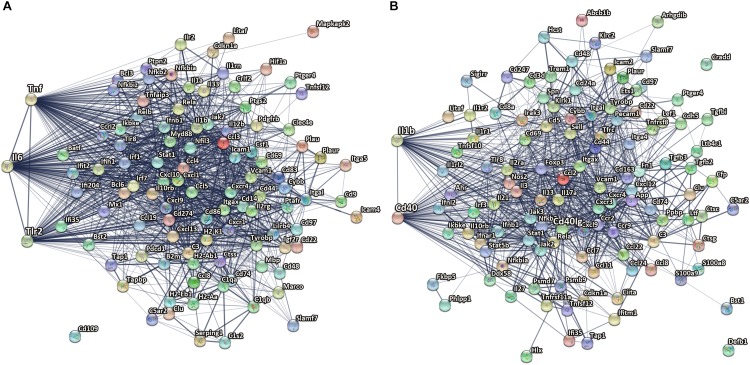
Microglial genes with highest confidence in gene networks after TBI. **(A)** TNF, IL6, and Tlr2 are among the top most confident centers of microglial upregulated genes network at 14 dpi. **(B)** IL-1β and CD40 are among the top 10 upregulated genes with the highest confidence in microglial gene networks at 60 dpi. The edge thickness indicates the strength of data support by STRING analysis, from thin, medium to thick corresponds to confident score 0.4, 0.7, and 0.9. The nodes are colored in alphabetical order of protein name, graphs in each node are known or predicted 3D structure.

At 14 dpi, TNF (direct association of 82 out of 102 genes), IL6 (direct association of 78 out of 102 genes), and Toll-like receptor-2 (direct association of 62 out of 102) were among the top most confident centers of microglial upregulated gene networks. These findings are consistent with studies showing a critical role for IL 6 (Poulsen et al., 2005), TNF (Longhi et al., 2013), and TLR-mediated signaling (Hua et al., 2011) pathways in induction and regulation of inflammatory responses in the acute stages following injury. Our study provides evidence of a role in the subacute stages post-injury, consistent with a prior study showing differential effects of TNF knockout on motor function early vs. late after CCI (Bermpohl et al., 2007; Figure 6A).

One of the top downregulated genes with the highest confidence in microglial gene networks at 14 dpi was CCR5. CCR5 is a cysteine–cysteine chemokine receptor which is highly expressed in microglia and is important for chemotaxis, *in vitro* and *in vivo* (Bajetto et al., 2002). Pharmacological blockade of CCR5 reduced migration velocity and the number of perilesional migratory microglia in a model of focal brain injury (Carbonell et al., 2005). Downregulation of CCR5 might impact microglia migration to injured brain regions at a subacute phase post insult. In addition, previous work used CCR2/5 inhibitor in aged animals following TBI showed significant reduction in the recruitment of peripheral macrophages to the injured site and decrease in their pro-inflammatory response (Morganti et al., 2016a). Another recent study used CCR5 knockdown showed improved cognitive recovery in a pre-clinical closed head injury TBI model and also associated with enhanced motor recovery after stroke in patients with mutated CCR5 gene (Joy et al., 2019).

At 60 dpi, CD40 is one of the upregulated genes with the highest confidence in the microglial gene networks with direct association of 59 of 112 genes. CD40 is a member of the tumor necrosis factor (TNF) receptor superfamily. CD40 is a receptor on antigen-presenting cells (including macrophages and microglia) and an essential mediator of various inflammatory responses (Elgueta et al., 2009). Abnormal expression of CD40 has been associated with autoimmune inflammatory diseases such as multiple sclerosis (Aarts et al., 2017), as well as AD pathology in experimental animals (Calingasan et al., 2002; Laporte et al., 2006). Furthermore, antagonizing CD40L and genetic disruption of CD40 signaling improved cognitive function and AD-related pathology in AD animal models (Tan et al., 2002; Laporte et al., 2006; Figure 6B).

In addition, IL-1β was also found to be among the top 10 upregulated genes with the highest confidence in microglial gene networks at 60 dpi. IL-1β is a pro-inflammatory cytokine that is upregulated as part of the acute inflammatory innate host response to trauma, infection, and other types of injury (Garlanda et al., 2013; Figure 6B). IL-1β is also known to be a key mediator of neuronal function (Smith et al., 2009), glial activation, and immune cell recruitment as well as neurodegeneration in CNS injury models and neurodegenerative diseases (Wang et al., 2015). IL-1β is acutely increased after TBI in humans and experimental TBI models, and blocking its activity has been shown to be beneficial in experimental models of TBI (Clausen et al., 2009, 2011; McKee and Lukens, 2016). The genetic antagonism of IL-1R1 had divergent effects on neurological function in focal vs. diffuse injury TBI models (Chung et al., 2019). In line with the literature we identified IL-1β to be a key regulator not only at acute and subacute time points but also at chronic stages (60 dpi) after CCI. The association between IL-1β chronic inflammatory profiles and long-term outcome post-injury should be further evaluated.

### Effects of Injury on the Microglial Biological Pathways

We performed GSEA (Online Methods) to identify pathways that are differentially changed in microglia post-injury. GSEA pathway analyses showed upregulation of multiple mechanisms in chronic time points post-injury including immune system, cytokine–cytokine receptor interaction, KEGG chemokine signaling, adaptive immune system, KEGG cell adhesion CAMs, JAK-STAT signaling pathways, and cytotoxicity. Interferon signaling, Toll receptor cascades, NOD-like receptor signaling, and IFN alfa and beta pathways were more upregulated at 14 dpi. These data constitute further evidence of a time-dependent injury-associated change in the microglial phenotype toward a pro-inflammatory state (Supplementary Figure S4).

### Relative Expressions of Selected Microglia Genes for Sham vs. 2 Dpi Were Measured by Real-Time qPCR

To validate our Nanostring gene expression analysis, RT^2^ qPCR was used to measure the relative expression of three upregulated genes including (TNF, IL1b, and IL6). Similar to the 2 dpi Nanostring data, there was significant upregulation of these three genes at 2 dpi when compared sham vs. injured mice (*n* = 3) (Supplementary Figure S5).

## Discussion

To our knowledge this is the first study to define the temporal course of microglial inflammation-related gene expression at acute, subacute, and chronic time points in a pre-clinical TBI model. We identified gene expression changes suggesting dysregulation of the ability of microglia to sense tissue damage, perform housekeeping functions, and maintain homeostasis in the early stages post-CCI, with subsequent recovery of these pathways over time. We also identified injury-associated changes in the microglial phenotype toward a chronic pro-inflammatory state post-TBI.

Microglia have extensive processes that allow them to detect changes in their environment. For this purpose, microglia also have an armamentarium of cellular receptors and proteins previously defined by our group as the “microglia sensome” (Hickman et al., 2013). In this dataset, we found downregulation of microglia sensome genes at 2 dpi followed by upregulation at later time points. Dysregulation of microglial sensome early after TBI has not been previously reported in a TBI model. Further studies are needed to assess the functional impact of sensome gene downregulation after CCI.

Our data also suggest that TGF-β cytokine signaling behaved in a pattern similar to sensome genes at 2 dpi. TGF-β is essential for maintaining a microglial homeostatic phenotype (Butovsky et al., 2014). The downregulation of TGF-β may in part regulate the reduced expression of microglial sensome genes at 2 dpi. TGFβ signaling is involved in regulating microglial activation and its disruption could potential lead to neuroinflammatory response dysregulation, increased cytotoxicity (Brionne et al., 2003), and it also has to be associated with many neurodegenerative diseases including AD (Town et al., 2008; von Bernhardi et al., 2015). The cross talk between TGF-β and microglia sensome post-injury has not been fully evaluated in TBI and given the key regulatory role of this signaling pathway, further studies are needed to evaluate the TGFβ-mediated effects on microglial functions and long-term outcome in TBI.

Of note, triggering receptor expressed on myeloid cells 2 (TREM2), an innate immune receptor expressed on the surface of microglia, was also significantly downregulated at 2 dpi compared to sham then returned to baseline expression by 14 dpi. TREM2 is also part of the microglial sensome and is expressed on dendritic cells, osteoclasts, and macrophages (Hickman et al., 2013). TREM2 forms a signaling complex with the adaptor tyrosine kinase-binding protein (TyroBP or DAP12) which in turn plays critical roles in regulating microglia activation, enhance their sensing functions, survival, phagocytosis, and cytokine production (Hickman and El Khoury, 2014). In addition, TREM2–DAP12 signaling plays a central role in the pathogenesis of several diseases (beneficial for some diseases and harmful for others) including frontotemporal dementia, AD, and multiple sclerosis (Hickman and El Khoury, 2014; Hickman et al., 2018). However, the role of TREM2–DAP12 signaling in TBI is still not yet known. Trem2 knockout mice had reduced macrophage infiltration at 3 dpi, and reduced hippocampal atrophy, and improved behavioral outcome at 120 dpi in a lateral fluid percussion model, suggesting a role for TREM2 in outcome after cerebral contusion (Saber et al., 2017). Further studies are warranted to further investigating the role of this key microglial regulator post-injury.

The proinflammatory IFNγ genes had a more complex response post-TBI. A large number of genes in this pathway were upregulated at 14 dpi. Interestingly, those genes returned to baseline at 60 dpi. Interestingly, genes in this pathway that remained unchanged at 14 dpi were upregulated at 60 dpi. IFNγ upregulates the neurotoxic pathways in microglia including reactive oxygen species (ROS) production and others, as well as genes involved in antigen presentation and proinflammatory T-lymphocyte-related chemokines (Takeuchi et al., 2006; Moran et al., 2007; Spencer et al., 2016). Similarly, CCI upregulated the anti-inflammatory cytokine pathways of IL-4 and IL-10 response genes at 14 and 60 dpi. Our data suggest there is a biphasic pattern of IFNγ, IL-4, and IL-10 gene expression changes, with concurrent proinflammatory and anti-inflammatory states at subacute and chronic timepoints post-TBI. This mixed inflammatory profile indicates that the oversimplified pro-inflammatory “M1”and anti-inflammatory “M2” polarization states do not necessarily reflect the diversity of microglia functions and their related signaling pathways during complex diseases such as TBI (Morganti et al., 2016b; Hirbec et al., 2017; Jassam et al., 2017).

Our study has some limitations; first, our sampling method using the entire brain to interrogate microglia gene expression is limited by inclusion of microglia that may not undergo significant morphological changes but may still perhaps undergo transcriptional changes even though distant from contusion. We think our methodology is the most non-biased, but we do recognize that we may lose some signal with this approach. Further single cell studies to evaluate the regional differences in microglia activation after injury are warranted. Second, sex-specific transcriptomic profiles were found when comparing adult female and male mice (Hanamsagar et al., 2017); however, we used only male mice in our study, and future studies in addressing the impact of gender on microglial gene expression at acute and chronic timepoints.

The generation of our transcriptomic data sets is an important step forward to understand microglia biology and functional diversity and their role in TBI pathogenesis at the molecular level and identify common pathways that affect outcome. However, because of the mixed picture noted in the subacute and chronic stage, we need to better understand cellular cross talk post-injury at the single cell level, taking into consideration age, gender, and anatomical proximity to the injury to establish definitive profiles for microglia during different stages of TBI. Recent, single-cell RNA-Seq to study the impact of concussive injury on single cell hippocampal cell types in the acute stage is a step in the right direction (Arneson et al., 2018). More studies to evaluate gene expression at the single cell level and in focusing on subacute and chronic timepoint are warranted. Establishing a definitive quantitative time-dependent microglia transcriptome post focal head injury will open the door for more precise experimentation using targeted gene deletion with Cre–Lox approach and inducible microglia promoter-specific gene manipulation to identify potential novel targets for treatment of TBI.

## Data Availability

The gene expression data have been uploaded to the GEO repository. Following is the associated link https://www.ncbi.nlm.nih.gov/geo/query/acc.cgi?acc=GSE132809.

## Ethics Statement

Experiments were performed and animals were treated humanely according to ARRIVE guidelines. All procedures were performed in accordance with the NIH Guide for Care and Use of Laboratory Animals and followed protocols approved by the MGH Institutional Animal Care and Use Committee.

## Author Contributions

SI, ZF, SL, SH, NP, DK, JK, and MW: manuscript conception and design. QL: equal contribution to the manuscript concept and design, acquisition of data, analysis and interpretation, and critical revision of the manuscript for important intellectual content. CP: acquisition and analysis of the data. SI, SL, LW, JC, AS-S, AB-W, SH, JK, and MW: acquisition of the data, and analysis and interpretation. QL, ZF, SL, JC, AS-S, NP, and DK: critical revision of the manuscript for important intellectual content. SI, LW, SH, JK, and MW: critical revision of the manuscript for important intellectual content and study supervision.

## Conflict of Interest Statement

The authors declare that the research was conducted in the absence of any commercial or financial relationships that could be construed as a potential conflict of interest.

## Footnotes

1 https://imagej.nih.gov/ij/

## Supplementary Material

FIGURE S1 Gating strategy for flow cytometry. Gates for sorting population of microglia (CD11b high and CD45 low to intermediate) are shown.

FIGURE S2 Sensitivity analysis of *p*-measurement by cutoff_*high*_ and cutoff_*low*_ in pattern-oriented time-series clustering. Higher *p*-measurement corresponds to less noise in clusters of genes.

FIGURE S3 REACTOM pathways of three clusters that follow a specific pattern over the three study time points. Pathway analysis of level of significance by –log10(FDR) of up-regulated genes in the three comparisons from left to right: stable-stable-up, stable-up-up, and stable-stable-down. Genes in the stable-stable-up cluster were involved in secondary injury mechanisms including cytokine receptor and cytokines interactions, chemokine receptors, and immune, adaptive immune system, and cell-mediated toxicity.

FIGURE S4 REACTOM pathways. Pathway analysis of level of significance by –log10(FDR) of up-regulated genes in the three comparisons from left to right: 2 dpi vs. sham, 14 dpi vs. sham, and 60 dpi vs. sham. This showed that the pathways are most significantly altered in 14 and 60 dpi.

FIGURE S5 Relative expressions of selected microglia genes for sham vs. 2 dpi were verified by real-time qPCR. Relative RNA expression of selected genes including TNF, IL1b, and IL6 RNA measured by PCR in sham vs. injured groups (*n* = 3) at 2 dpi. Consistent with the Nanostring data, there is significant upregulation of TNF, IL1b, and IL6 at 2 dpi in CCI injured vs. sham mice. Error bars indicate standard errors of the mean. ^*^*p* < 0.05.

TABLE S1 List of significantly changed genes at 2 dpi vs. sham.

TABLE S2 List of significantly changed genes at 14 dpi vs. sham.

TABLE S3 List of significantly changed genes at 60 dpi vs. sham.

TABLE S4 List of clusters and genes per cluster that follow a specific pattern over the three study time points.
