# Supplementary figures and images for: Time-Dependent Changes in Microglia Transcriptional Networks Following Traumatic Brain Injury
Source: Front Cell Neurosci. 2019 Aug 8;13:307. doi: 10.3389/fncel.2019.00307 (PMC6694299; doi:10.3389/fncel.2019.00307)

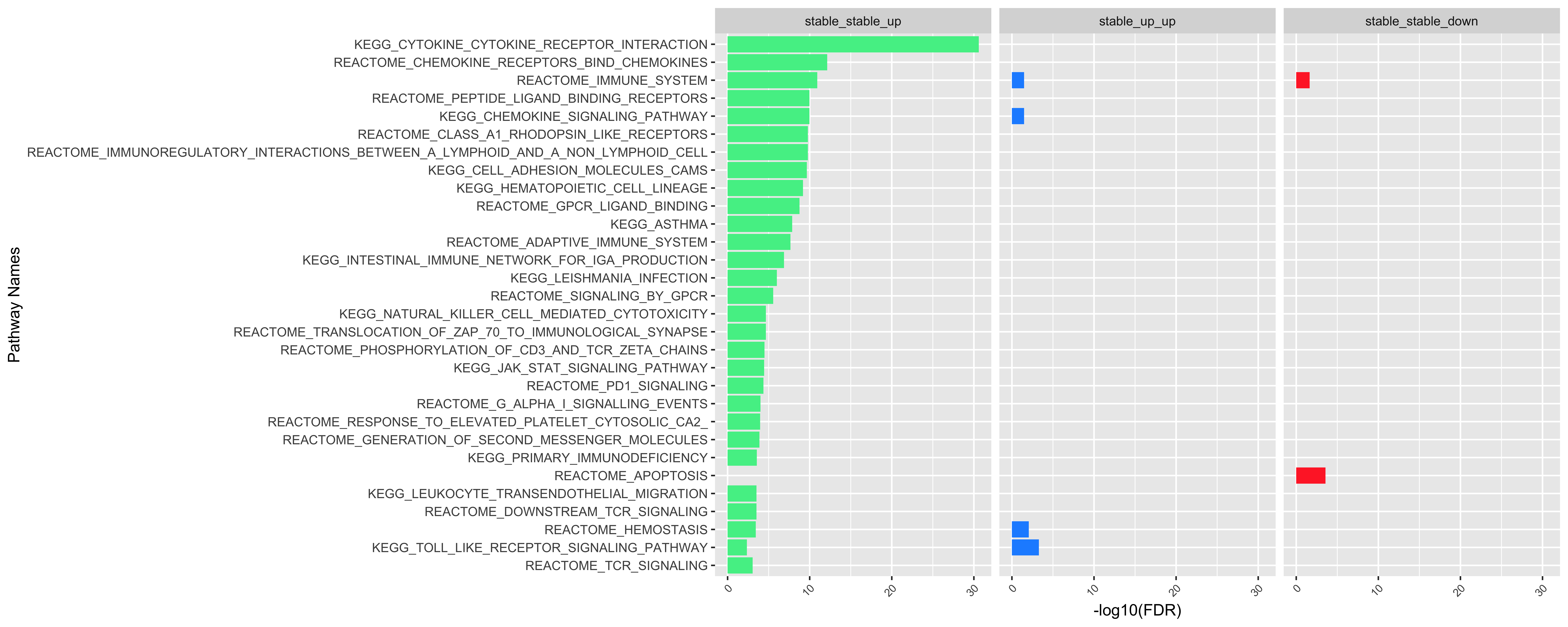

Supplement: FIGURE S1 — Gating strategy for flow cytometry. Gates for sorting population of microglia (CD11b high and CD45 low to intermediate) are shown. [file Data_Sheet_1.zip › Supplementary Material/Supplemental Figure 3.png]

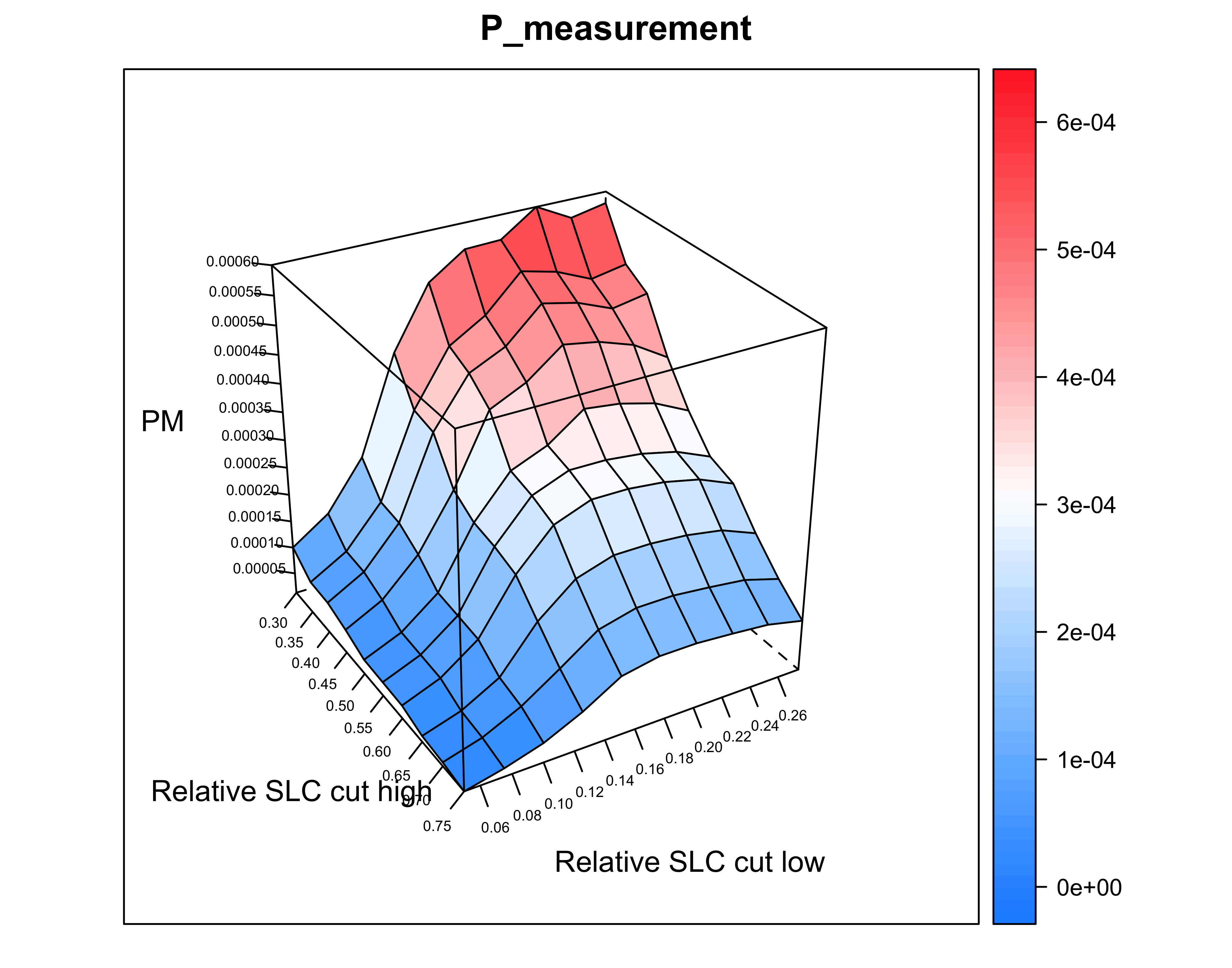

Supplement: FIGURE S1 — Gating strategy for flow cytometry. Gates for sorting population of microglia (CD11b high and CD45 low to intermediate) are shown. [file Data_Sheet_1.zip › Supplementary Material/Supplementary Figure 2. Sensitivity analysis.png]

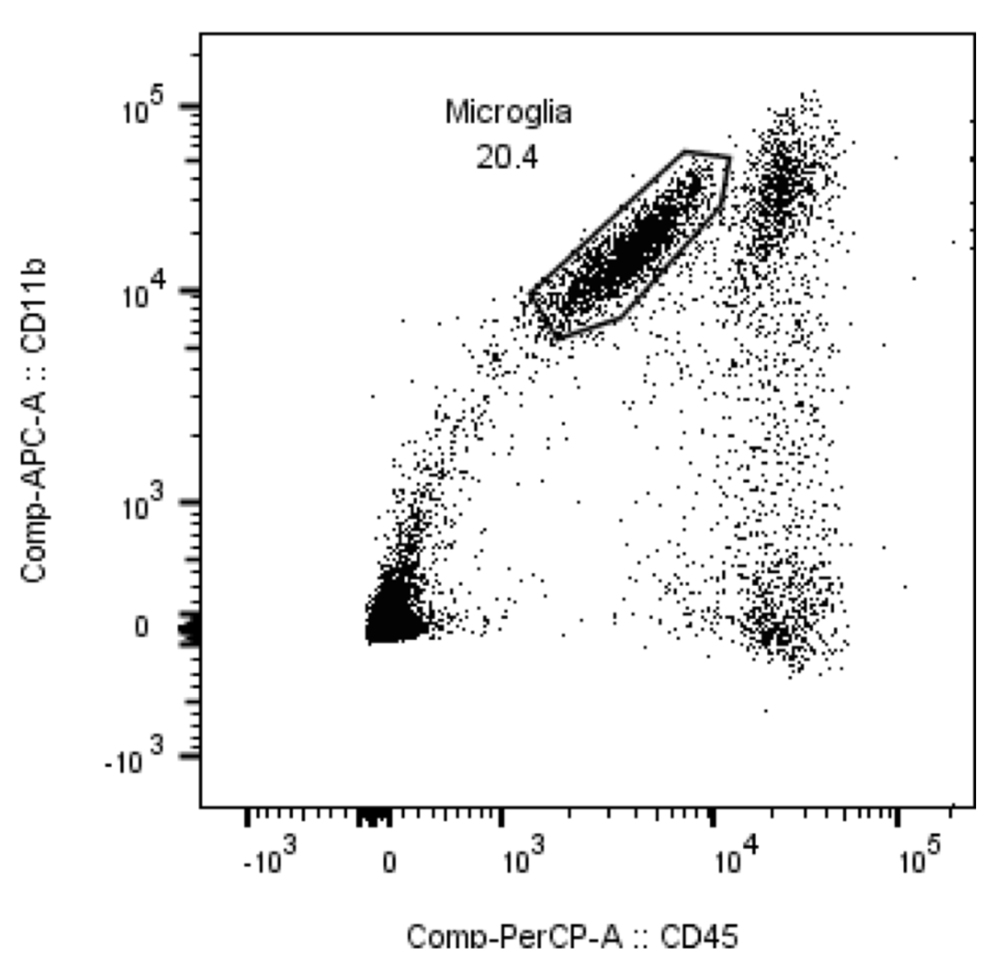

Supplement: FIGURE S1 — Gating strategy for flow cytometry. Gates for sorting population of microglia (CD11b high and CD45 low to intermediate) are shown. [file Data_Sheet_1.zip › Supplementary Material/Supplemental Figure-1.jpg]

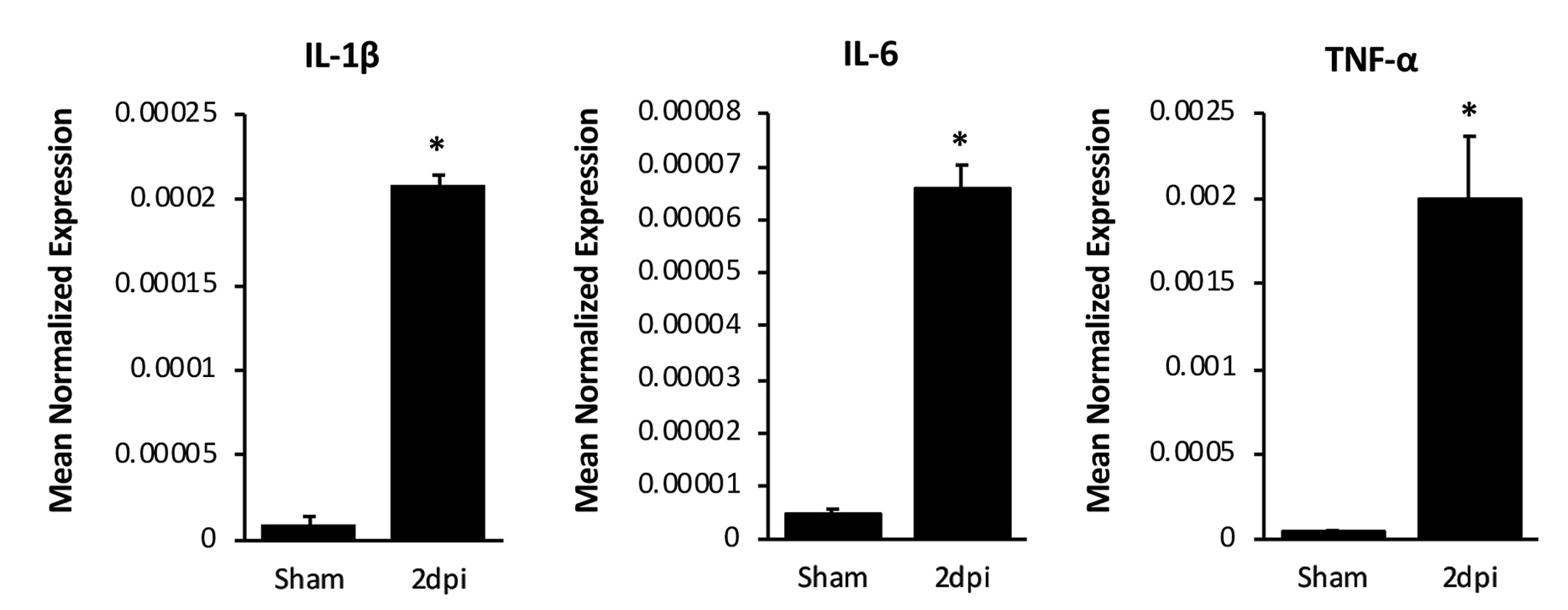

Supplement: FIGURE S1 — Gating strategy for flow cytometry. Gates for sorting population of microglia (CD11b high and CD45 low to intermediate) are shown. [file Data_Sheet_1.zip › Supplementary Material/Supplemental Figure 5.jpg]

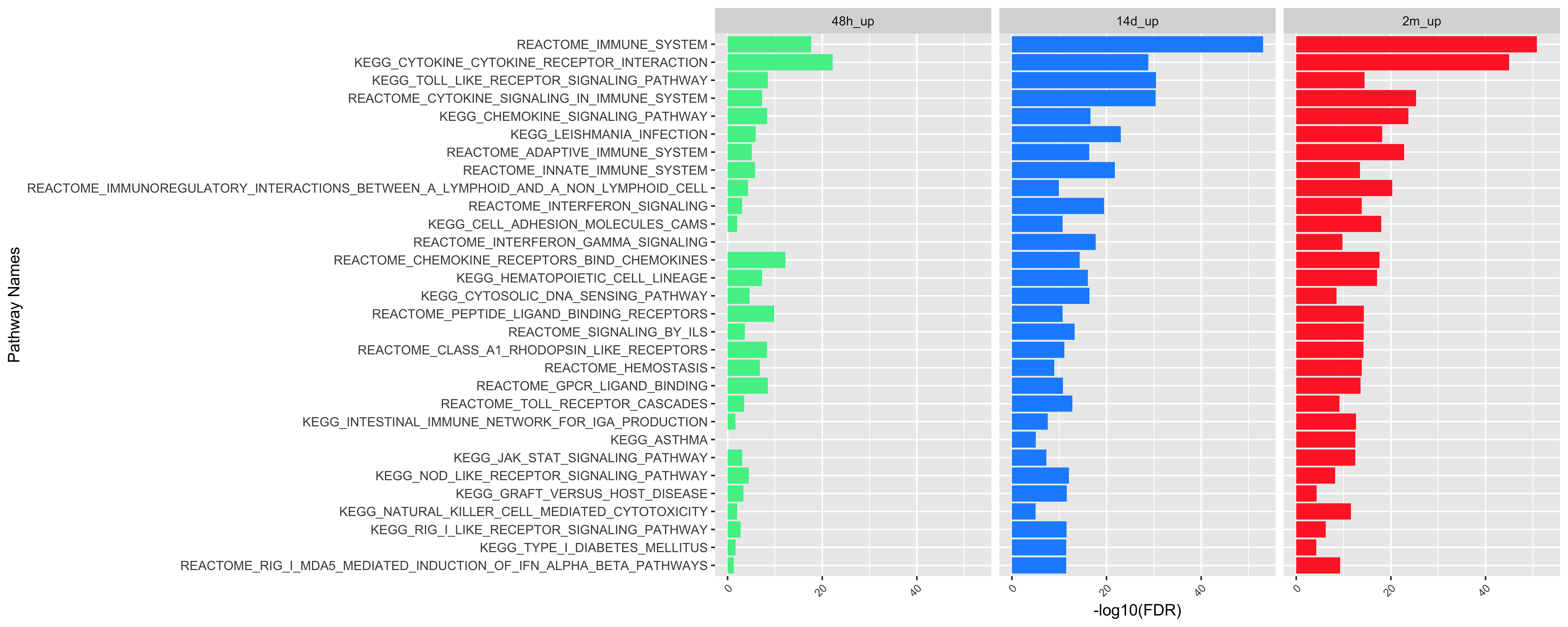

Supplement: FIGURE S1 — Gating strategy for flow cytometry. Gates for sorting population of microglia (CD11b high and CD45 low to intermediate) are shown. [file Data_Sheet_1.zip › Supplementary Material/Supplemental Figure 4.png]
